# Random mutagenesis of the hyperthermophilic archaeon *Pyrococcus furiosus* using *in vitro* mariner transposition and natural transformation

**DOI:** 10.1038/srep36711

**Published:** 2016-11-08

**Authors:** Natalia Guschinskaya, Romain Brunel, Maxime Tourte, Gina L. Lipscomb, Michael W. W. Adams, Philippe Oger, Xavier Charpentier

**Affiliations:** 1 Univ Lyon, Université Claude Bernard Lyon 1, INSA-Lyon, CNRS, UMR5240, Microbiologie, Adaptation et Pathogénie, 10 rue Raphaël Dubois, F-69622, Villeurbanne, France; 2 CIRI, Centre International de Recherche en Infectiologie, Inserm, U1111, Université Claude Bernard Lyon 1, CNRS, UMR5308, École Normale Supérieure de Lyon, Univ Lyon, 69100, Villeurbanne, France; 3 Univ Lyon, ENS de Lyon, CNRS UMR 5276, Lyon, France; 4 Univ Lyon, INSA-Lyon, Université Claude Bernard Lyon 1, CNRS, UMR5240, Microbiologie, Adaptation et Pathogénie, 11 Avenuue Jean Capelle, 69621 Villeurbanne cedex, France; 5 Department of Biochemistry and Molecular Biology, University of Georgia, Athens, Georgia USA

## Abstract

Transposition mutagenesis is a powerful tool to identify the function of genes, reveal essential genes and generally to unravel the genetic basis of living organisms. However, transposon-mediated mutagenesis has only been successfully applied to a limited number of archaeal species and has never been reported in *Thermococcales*. Here, we report random insertion mutagenesis in the hyperthermophilic archaeon *Pyrococcus furiosus*. The strategy takes advantage of the natural transformability of derivatives of the *P. furiosus* COM1 strain and of *in vitro* Mariner-based transposition. A transposon bearing a genetic marker is randomly transposed *in vitro* in genomic DNA that is then used for natural transformation of *P. furiosus.* A small-scale transposition reaction routinely generates several hundred and up to two thousands transformants. Southern analysis and sequencing showed that the obtained mutants contain a single and random genomic insertion. Polyploidy has been reported in *Thermococcales* and *P. furiosus* is suspected of being polyploid. Yet, about half of the mutants obtained on the first selection are homozygous for the transposon insertion. Two rounds of isolation on selective medium were sufficient to obtain gene conversion in initially heterozygous mutants. This transposition mutagenesis strategy will greatly facilitate functional exploration of the *Thermococcales* genomes.

It is now well established that Archaea represent a taxonomic domain distinct from Bacteria, their prokaryotic counterpart. Recent phylogenetic analysis even suggest a Bacteria-Archaea two domain tree of life in which Eukaryotes found their origin within the Archaeal domain^1^,^2^. Even early in the discovery of Archaea, phenotypic properties distinguished them from Bacteria while suggesting similarity with eukaryotes. Indeed, Archaea were found to be insensitive to most 70S-targeted drugs such as streptomycin, but were sensitive to 80S-targeted drugs such as anisomycin^3^. Further studies confirmed that Archaea shared many features with eukarya, including their ribosomal proteins^4^. From a practical standpoint, isolated archaeal macromolecular systems, such as the ribosome, are simpler and easier to study than their eucaryotic counterparts. Thus, Archaea have allowed faster understanding of many eucaryal processes^4^. Far from being simplistic versions of eukaryotes, Archaea have very unique features, linked to their ability to thrive at the extremes of pH, temperature, salinity and hydrostatic pressures. These properties are biologically and biochemically intriguing and makes Archaea one of the most important resources for biotechnological and industrial applications^5^,^6^. However, this resource remains difficult to access even in the age of genomics. Archaeal genomes are predominantly annotated from the knowledge of bacterial and eukaryal processes. As a result, there is a high fraction of genes for which no biochemical function can be assigned, and for instance can exceed 60% in some families such as the Thermococcales^7^,^8^,^9^.

Functional exploration of archaeal organisms remains limited by the lack of proper genetic tools. This is particularly evident in extremophiles whose extreme growth conditions prevent the use of genetic tools developed in mesophilic organisms and also render commonly used selective agents ineffective^10^. The discovery of the first archaeal plasmids allowed a first major step in the development of tools for archaeal genetics, and to date, several replicative plasmids, expression vectors, and transformation procedures are available for the major groups of Archaea^11^. Yet, few methods are currently available to generate random mutagenesis and identify genes of interest in uncharacterised biological processes by forward genetic screens. To date, only four archaeal species are amenable to random mutagenesis, and all are mesophilic species. The first two systems were developed for the halophile *Haloarcula hispanica*^12^,^13^ and the methanogen *Methanosarcina acetivorans*^14^ by engineering a native insertion sequence or the insect transposon Mariner, respectively. More recently, *Methanococcus maripaludis*, another methanogen, was mutagenised very efficiently using a Tn5 derivative by transforming the cells with a mixture of the transposon and the transposase^15^. The last system is based on Mu *in vitro* transposition followed by the transfer of the mutagenised fragments into the moderate halophile *Haloferax volcanii* genome by chemical transformation^16^. Thus, to date, no random mutagenesis approach exists for thermophilic archaea.

*In vitro* transposition has a great potential for random mutagenesis of the most extremophilic archaea since it does not require a transposon to function in the species to be mutagenised. However, the delivery of DNA in archaea which relies on chemical transformation, electroporation, spheroplast transformation, liposomes or conjugation with *Escherichia coli* is often very inefficient^11^. As a consequence, obtaining a large library of mutants may be difficult. In some archaeal species, DNA uptake is greatly facilitated by the ability to spontaneously take up extracellular DNA, allowing integration into the genome and subsequent genetic transformation. This natural transformation occurs at low frequencies in methanogens^17^,^18^, acidophiles^19^ and in the hyperthermophiles *Thermococcus kodakarensis*^20^, *T. barophilus*^21^ and *Pyrococcus furiosus*^22^. In this latter species, a genetically tractable variant named COM1 shows remarkable natural competence with transformation frequencies reaching 10^3^ transformants per μg of DNA^22^. COM1 is also an uracil auxotroph due to the deletion of the PF1114 gene (Δ*pyrF*)^22^,^23^ which permits the efficient selection of transformants on selective medium without uracil, using the wild-type PF1114 gene as a selection marker. Site-specific integration of the uracil prototrophy marker can be obtained if its sequence is synthetically flanked with the targeted sequence. This strategy has largely been exploited to generate insertion or deletion mutants of *P. furiosus* in a single step^22^,^23^,^24^. Thus, uracil prototrophy selection, combined with the efficient natural transformation of COM1 allows the possibility to obtain targeted mutants in large numbers. With the random insertion of the uracil protophy marker into genomic DNA, natural transformability of COM1 with this mutagenized DNA could generate libraries of random mutants.

In this study, we describe the development of an approach to generate a random transposon insertion mutant library for *Pyrococcus furiosus*. This approach combined two steps: first, *in vitro* transposition mutagenesis of the genomic DNA of *P. furiosus* using a *mariner*-based system to generate a library of transposons inserted at random DNA loci; and second, the transfer of the randomly mutated loci to the *P. furiosus* chromosome by natural transformation. The high natural transformation frequencies allowed us to generate libraries of several thousand mutants of *P. furiosus*. Interestingly, this procedure has the potential to be adapted to other archaeal species.

## Results and Discussion

### Parameters affecting transformation and selection of transformants on solid medium

**Figure 1:**
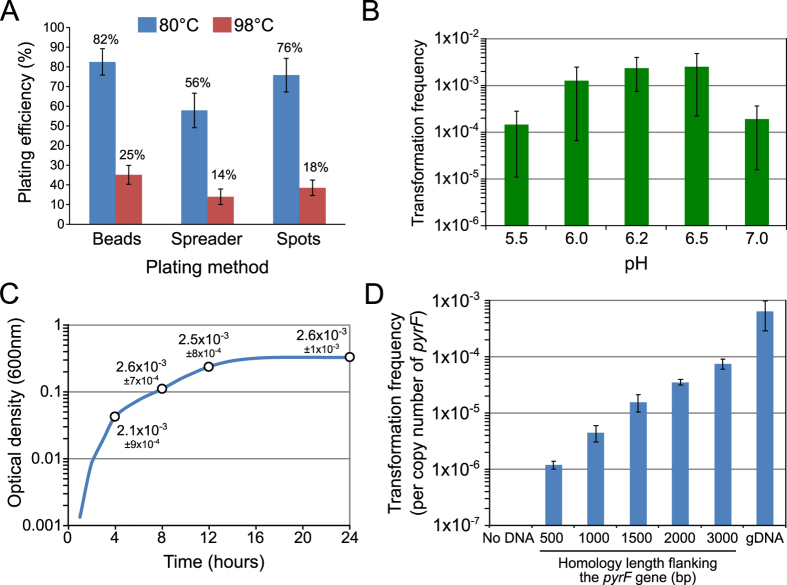
Growth and transformation conditions of *P. furiosus* COM1. (**A**) Plating efficiencies using glass beads, plastic spreader and spotting at two temperatures of incubation (80 °C and 98 °C). Error bars indicate standart deviation from the mean of three independent experiments. (**B**) Transformation frequencies as a function of pH using flanking regions of 1500 bp. Transformation reactions contained 2 μg DNA per 1 ml of culture. 100 μl or 200 μl of cell culture (10^8^ cells/ml) were respectively mixed with 200 ng or 400 ng of DNA. The transformation frequencies are calculated as the ratio of CFUs on selective medium (without uracil) divided by CFUs on non-selective medium (with uracil) per μg of DNA. Error bars represent standard deviation from the mean of eight independent experiments. (**C**) Transformation frequencies as a function of growth phase. Growth was monitored every two hours for 24 hours. Cells collected from a culture at time corresponding to exponential growth (4 h), mid-log (8 h), post-exponential (12 h) and stationary phase (24 h) were used for transformation in the same conditions as in panel B. Errors represent standard deviation from the mean of three technical replicates. (**D**) Transformation frequencies as a function of the size of the homologous flanking regions. Frequencies have been normalised to the copy number of the *pyrF* gene. Error bars indicate standart deviation from the mean of five independent experiments.

In order to maximise efficiency in construction of a random mutagenesis library for *P. furiosus*, we first sought to optimise conditions to increase the efficiency of the transformation experiments. Selection of transformants of *P. furiosus* by natural transformation requires growth on minimal solid medium^22^. This defined minimal medium is plagued by a relatively low plating efficiency with only 20% of cells forming colonies^25^. This minimal defined medium contains cellobiose and amino acids as carbon sources, and at elevated temperatures, these can react to form Maillard reaction products that are inhibitory to archaeal growth^26^. The formation of brown coloured Maillard reaction products is highly dependent on temperature. Indeed, there is a marked change in colour of the growth medium after incubation. The higher the temperature, the darker the colour, and the slower the growth of *P. furiosus*. Since *P. furiosus* can grow from 70 to 103 °C^27^, we have evaluated the plating efficiency at 98 °C compared to 80 °C, a temperature low enough to reduce Maillard reaction products but still sufficient to allow growth of *P. furiosus*, albeit at a slower rate (doubling time of ~2 h vs ~1 h). We also evaluated three different methods of plating: glass beads, a plastic spreader or direct spotting of liquid cultures on the plates. The plating techniques have limited impact on the plating efficiencies at 98 °C, and range from 15% when plating with a plastic spreader to 25% when plating with glass beads (Fig. 1A). These relatively low plating efficiencies are comparable to previous reports^22^,^25^,^28^. In contrast, we achieved plating efficiencies of up to 82% by incubating plates at 80 °C (Fig. 1A) and spreading with glass beads. For each plating method, we observed a 3 to 4-fold improvement at 80 °C compared to the high temperature incubation. This is further proof that the occurrence of the Maillard reaction, evidenced by the browning of the growth medium, is likely to be responsible for the low plating efficiencies on minimal medium at 98 °C. In subsequent experiments plating was performed using glass beads or spotting, and incubating the plates at 80 °C. We then assessed if natural transformation frequencies could be affected by abiotic or biotic parameters known to impact natural transformation in bacteria, such as pH and growth phase. We found that transformation frequencies varied by more than an order of magnitude with pH, with an optimum at pH 6.2 to 6.5 (Fig. 1B). For some naturally competent bacteria, growth phase can play a large role in natural transformation; however, transformation frequencies of *P. furiosus* were not dependent on growth phase (Fig. 1C). Thus, the maximum number of transformants of *P. furiosus* COM1 can be obtained by transforming overnight stationary-phase cultures, selecting on plates at pH 6.5, and incubating at 80 °C. All experiments presented hereafter have been performed using these conditions.

### Genomic DNA is the most effective molecule for transformation of *P. furiosus*

*P. furiosus* strain COM1 is naturally competent for uptake of genomic, circular and linear DNA^22^,^23^. The recombination efficiency in *P. furiosus* COM1 allows transformation with the *pyrF* gene with very short homology regions, as small as 40 bp^23^. Longer homology regions up to 1 kb increased the number of obtained transformants by three orders of magnitude^23^. To generate a large number of random mutants, it was crucial to determine the type and size of DNA molecule that was most efficient in transformation experiments. This DNA molecule would subsequently become the target of *in vitro* random mutagenesis. To do so, we compared the efficiencies of the *pyrF* gene, either carried on PCR products or on genomic DNA (gDNA) to generate transformants by complementing the *pyrF* deletion in COM1. On PCR products, we varied the size of the homologous regions flanking the *pyrF* selectable marker starting from 500 bp to 3 kb. The length of these homologous regions are maximal when *pyrF* is carried on gDNA. As expected from the literature, the number of transformants obtained with PCR products increased with the length of homology, reaching several thousands transformants with only 200 ng of DNA mixed with 100 μl of cell suspension (~10^7^ cells). Interestingly, the best transformation frequencies were obtained with gDNA (Fig. 1D), allowing us to generate several thousand mutants per μg of gDNA. The efficient transformation of *P. furiosus* cells with gDNA is further indication that DNA is indeed taken up and integrated in the genome by an active natural transformation process. Furthermore, it indicates that gDNA represents the best substrate for *in vitro* random mutagenesis of this species.

### *In vitro* transposition of genomic DNA and transformation in *P. furiosus*.

**Figure 2:**
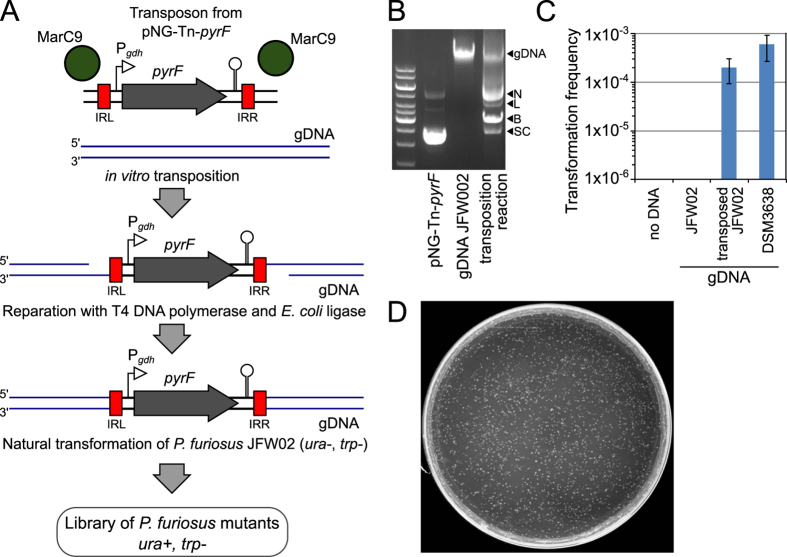
*In vitro* transposition of *mariner*-based vector containing the *Pgdh-pyrF* cassette into genomic DNA using the MarC9 transposase. (**A**) General strategy for the creation of a *P. furiosus* mutant library using *in vitro* transposition mutagenesis. The transposase MarC9 (dark green circles) recognises the IRL/IRR sites (in red) at the borders of P*gdh-pyrF* cassette (carried by the pNG-Tn-*pyrF* plasmid) and transposes it by a cut-and-paste mechanism into genomic DNA (gDNA). Transposed gDNA is repaired using T4 DNA polymerase and *E. coli* DNA ligase and used to transform the uracil and trytophan auxotroph JFW02. (**B**) Agarose gel electrophoresis of the *in vitro* transposition reaction. Lane 1, transposon donor plasmid pGN-Tn-pyrF alone; lane 2, genomic DNA of strain JFW002 alone; lane 3, transposition reaction containing genomic DNA, donor plasmid pGN-Tn-pyrF and MarC9 transposase. Excision of the transposon from the transposon-donor plasmid pNG-Tn-*pyrF* can be evidenced by the appearance of a band at the size of the linear plasmid lacking the transposon (N, nicked plasmid; L, linear plasmid; B, backbone plasmid with transposon excised; SC, supercoiled plasmid). **(C**) Transformation frequencies of *P. furiosus* JFW002 transformed with *in vitro* transposed gDNA. Error bars represent standard deviation from the mean of four independent experiments. (**D**) Example of a transformation reaction with gDNA plated on a selective plate of minimal medium and incubated 5 days at 80 °C.

The natural transformability of *P. furiosus* with genomic DNA offers the possibility to implement an *in vitro* random transposon mutagenesis strategy as previously described for naturally transformable bacteria^29^,^30^. In this strategy genomic DNA is subjected to *in vitro* transposon mutagenesis with the hyperactive form of the *mariner* Himar1 transposase (MarC9)^31^ and a suitable mini-transposon. The mutagenised gDNA is repaired and used to transform the naturally competent species (Fig. 2A). To implement this strategy in *P. furiosus* we constructed a transposon-containing vector (pNG-Tn-*pyrF*) with a selectable marker (P*gdh-pyrF*) flanked by two inverted tandem repeat sequences (IRL, IRR) specific for the MarC9 Himar1 transposase (Fig. 2A and Supplementary Fig. S1). In order to ensure that transposon insertion effectively disrupts the open-reading frame, stop codons in all possible reading frames were added next to the inverted repeats. The transposon contains a wild type copy of the *pyrF* gene under the transcriptional control of the glutamate dehydrogenase promoter (*gdh*; PF1602) with a short terminator sequence (T1) from the *hpyA1* gene at the 3′ end of the gene^22^,^23^ (Fig. 2A). The hyperactive MarC9 Himar1 transposase recognizes the IRL and IRR sites and transposes intervening DNA by a cut-and-paste mechanism into target DNA at TA sites^32^. When incubated with the transposon-containing plasmid pNG-Tn-*pyrF*, the transposase cuts out the transposon, leaving out the plasmid backbone and randomly transposes it into the supplied gDNA (Fig. 2B). The transposition event leaves single-stranded gaps at each transposon-chromosome junction, and these are repaired using T4 DNA polymerase and *E. coli* DNA ligase (Fig. 2A,B). For the purposes of this application, it was important to use target genomic DNA isolated from a uracil auxotrophic *P. furiosus* strain that lacked the *pyrF* marker utilised in the transposon. Furthermore, to enable the use of the resulting library for other downstream applications that might require a second genetic marker, we chose to use *P. furiosus* strain JFW002 that is also a tryptophan auxotroph, containing a deletion in tryptophan biosynthetic genes (*trpAB*)^23^. JFW002 (COM1 Δ*pyrF* Δ*trpAB*) genomic DNA was transposed *in vitro* using the transposon-containing plasmid pNG-Tn-*pyrF* and the purified MarC9 Himar1 Mariner transposase (Fig. 2B). Transposed genomic DNA was then used to transform JFW002 cells using uracil prototrophy for selection of the *pyrF* marker. Compared to genomic DNA which carries *pyrF* at its original location, the transposon-mutagenised genomic DNA with randomly inserted *pyrF* gave rise to transformants with only slightly lower transformation frequency (Fig. 2C). A single transformation experiment, using 2 to 4 mL of culture and 2 to 4 μg of transposed DNA, generated from 700 to 2400 transformants (Fig. 2D). Combining four experiments we obtained a library of about 5000 mutants. Thus, *P. furiosus* can be efficiently and reproducibly transformed with genomic DNA that has undergone *in vitro* transposon-mediated mutagenesis.

### Natural transformation of *P. furiosus* with *in vitro* mutagenised genomic DNA generates random insertion mutants

**Figure 3:**
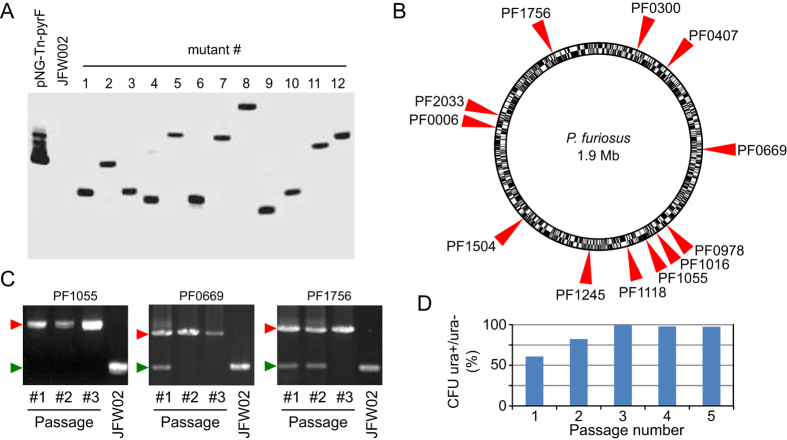
Identification and dynamics of transposon insertions in the genome of *P. furiosus* JFW002. (**A**) Southern blot analysis of 12 isolated and purified mutants probed with the *pyrF* gene. Lanes 1–12: purified randomly selected transposon insertion mutants; pNG-Tn-*pyrF*: positive control; JFW002: negative control. (**B**) COM1 genome distribution of the transposon insertions. Gene names (PFxxxx) according to the gene nomenclature for *P. furiosus* DSM 3638. (**C**) Typical gene conversion dynamics of transposon insertions monitored by PCR (known insertion). Due to meroploidy, all genomes of the mutant cells may not contain a mutated allele (red arrow, mutated allele; green arrow, wild-type allele). Conversion may require up to three successive subcultures in liquid selective medium. (**D**) Conversion dynamics of a pool of mutants assessed by plating on minimal medium with or without uracil. Mutants in which gene conversion is incomplete could generate uracil-auxotrophic progeny when plated on non-selective medium (ura+). A ratio of 100% ura^+^ CFU and ura^−^ CFU indicates that all genomes copies of cells contain the *pyrF* transposon.

In order to investigate the nature and dynamics of transposon insertions, we purified 12 mutants and confirmed the presence of the P*gdh-pyrF* cassette by PCR amplification. To verify the presence and the copy number of the transposon, these mutants were analysed by Southern hybridisation using a probe specific to the transposon (Fig. 3A). A single predominant fragment was detected in all of isolates, indicating that each harbored a unique transposon insertion. Moreover, each of the 12 tested mutants presented a specific size of hybridisation fragment, suggesting random transposition into the genome of *P. furiosus* JFW02 (Fig. 3A). The sites of transposon insertion were identified by single primer colony PCR^33^. We observed that all insertion events occurred at TA sites, and their position are located throughout the genome of *P. furiosus* JFW02 (Fig. 3B). In all the 12 investigated mutants the transposon interrupted an open reading frame indicating the non-essential nature of the corresponding genes (Table 1 and Supplementary Fig. S2).

Polyploidy has been suggested as a common trait of all Euryarchaeota^34^. The chromosome copy number in the *Thermococcales* family member *T. kodakarensis* has been determined to range from 9 to 17 copies^35^. Genome copy number has not been experimentally determined in *P. furiosus*, but being a closely related species to *T. kodakarensis*, we anticipated *P. furiosus* to also be polyploid. Thus, natural transformation of *P. furiosus* with randomly transposed DNA may initially generate a merodiploidy situation where one copy of a gene is inactivated, while other alleles remain wild-type. During the first few cell generations following transformation with transposed DNA, gene conversion may occur if the selection pressure is maintained, resulting in a single mutated allele in all chromosomal copies. We examined the dynamics of gene conversion following initial isolation of transposition mutants on selective medium. Using PCR analysis we found that 6 out of the 12 tested mutants already displayed only the mutated allele at the initial colony stage. For the remaining mutants two to three passages in selective liquid medium were sufficient to obtain gene conversion (Fig. 3C). Interestingly, mutants in which gene conversion is incomplete could generate uracil-auxotrophic progeny when plated on non-selective medium. We used this phenotype to assess gene conversion of a pool of mutants, which cannot be assessed by the PCR based techniques used for isolated mutants, and found that three passages in liquid selective medium were sufficient to increase the percentage of cells unable to generate uracil-auxotrophic progeny from 60% to nearly 100% (Fig. 3D). This is consistent with conversion rates observed in other archaeal species^34^,^36^,^37^,^38^.

## Conclusions

We report here that the highly efficient natural competence of the hyperthermophilic archaeon *P. furiosus* can be exploited to easily and rapidly obtain thousands of mutants for the generation of a random mutant library. The number of mutants obtained is dependent on both the plating efficiency and parameters increasing the efficiency of natural transformation, such as incubation temperature and pH. This technique should allow the establishment of an ordered collection of insertional mutants. Combined with a phenotypic screen, efficient random mutagenesis in *P. furiosus* will allow the identification of novel pathways and specific functions, as has been done for decades in Bacteria and Eucarya. Alternatively, a scale-up of the protocol would allow construction of a saturating insertional mutagenesis library that could be used to define essential and non-essential genes of *P. furiosus*. Furthermore, the method presented here could be applied to other archaeal species, with only one restriction, *e.g.* the existence of an efficient transformation protocol. Natural transformation presents several advantages as it does not require chemical or physical alteration of the cells and is efficient with high molecular weight intact gDNA. However, in species that do not efficiently take up gDNA naturally, *in vitro* mutagenesis could also be performed on fragmented gDNA and then delivered to cells through other methods of transformation.

## Materials and Methods

### Strains, media and growth conditions

The strains used in this study are listed in Supplementary Table S1. Plasmids used in this study are listed in Supplementary Table S2. The strains were grown anaerobically in a defined medium with cellobiose^25^ as the carbon source and supplemented with 20 μM uracil for COM1 and JFW002 strains^22^. Medium was prepared as previously described^22^. Liquid cultures were inoculated with a 1 to 2% inoculum or with a single colony in 5–50 ml of medium in 50–100 ml serum bottles degassed with three cycles of vacuum and nitrogen, reduced by addition of Na_2_S (0.1% final concentration), and incubated at 98 °C. Solid medium was prepared by mixing as 50:50 of liquid medium at 2X concentration with 1.6% (wt/vol) Gelrite® (SERVA) solubilised by boiling in water; both solutions were maintained at 95 °C just prior to mixing. The medium was poured into glass petri dishes immediately after mixing. The plates were poured and inoculated aerobically and then put in stainless steel anaerobic jars (Schuett Biotec) with one sachet of Gaspak® EZ Anaerobe (Becton Dickinson and Company, U.S.A). These sachets generate anaerobic conditions in the jar by elimination of O_2_ in less than 2 hours and production of CO_2_ in less than 24 hours. The jars were incubated for 4 days at 98 °C or 6 days at 80 °C. To test the effect of pH on the transformation frequency, the defined medium with cellobiose at 2X concentration was prepared by mixing of all components and adjustment to the indicated pH with 1 M NaOH or 1 M HCl. Purification of intermediate strains was performed by plating 10^−3^ dilutions of transformant cultures onto selective plate medium (without uracil) and picking isolated colonies into selective liquid medium.

### Plating efficiency

The density of *P. furiosus* overnight cultures was determined by counting in a Thoma counting chamber. To estimate the plating efficiency and cell viability, the 10^−3^, 10^−4^ and 10^−5^ dilutions of initial culture were plated using a plastic spreader or glass beads, or by spotting 10 μL of the dilutions. Plating efficiency was calculated as the ratio of the number of colonies observed on the plates to total cell counts determined by counting in a Thoma chamber.

### Transformation of *P. furiosus* COM1

For natural transformation, aliquots of *P. furiosus* culture, typically grown to mid-log phase (~10^8^ cells/ml, as determined by counting in a Thoma counting chamber) in defined liquid medium at pH 6.5 (except for experiment of Fig. 1B where various pH were tested), were mixed with DNA to obtain a final concentration of 2 μg/ml, as described previously^22^. Routinely, we used 100 μl or 200 μl of cell culture (10^8 ^cells/ml) mixed with 200 ng or 400 ng of DNA, respectively. The mixtures were plated on the selective solid medium without uracil using glass beads. Ten-fold serial dilutions were prepared in 1X base salts^22^ and spotted on the selective (using 10^−1^ and 10^−2^ dilutions) and non-selective plates (using 10^−3^, 10^−4^ and 10^−5^ dilutions) in order to calculate transformation frequencies. The plates were placed inverted in anaerobic jars and incubated at 80 °C for 5–6 days or at 98 °C for 3–4 days. The transformation frequency was calculated as the ratio of the number of CFUs counted on selective medium (without uracil) divided by the number of CFUs counted on non-selective medium (with uracil). DNA constructs used as substrates for natural transformation were produced by PCR amplification of the *pyrF* gene from wild-type gDNA using different lengths of homology regions ranging from 500 bp to 3000 bp, amplified using the following primer sets: pyrF_500F/pyrF_500R, pyrF_1000F/pyrF_1000R, GL061/GL062^22^, pyrF_2000F/pyrF_2000R, pyrF_3000F/pyrF_3000R (Supplementary Table S3).

### Genomic DNA isolation

Cells from 2 ml of overnight *P. furiosus* culture were harvested and suspended in 100 μl buffer A (25% sucrose, 50 mM Tris-HCl, 40 mM EDTA, pH 7.4). Next, 250 μl 6 M guanidinium HCl–20 mM Tris, pH 8.5 was added, and the mixtures were incubated at 70 °C for 5 min. Genomic DNA was extracted with phenol-chloroform-isoamyl alcohol (25:24:1; buffered at pH 8), ethanol precipitated, and suspended in 50 μl 10 mM Tris buffer, pH 8.0. Alternatively, genomic DNA was purified from 5 ml of overnight culture using the Promega Wizard® Genomic DNA Purification Kit per the manufacturer’s instructions.

### Construction of pNG-Tn-*pyrF*, plasmid with transposon for *in vitro* transposition.

The P*gdh-pyrF* cassette, containing 283-bp portion of the intergenic region upstream of *gdh* (PF1602) joined with *pyrF* gene and T1 terminator from the histone gene *hpyA1* (PF1722), was amplified from pJFW070^23^ plasmid DNA using primers Tn_pyrF_for (containing IRL-sequence and a sequence carrying stop codons in all 6 possible reading frames) and Tn_pyrF_rev (containing IRR-sequence and a sequence carrying stop codons in all 6 possible reading frames). The PCR product was purified and cloned in pJET1.2/blunt vector using CloneJET PCR Cloning Kit following the manufacturer’s instructions (Thermo Scientific, #K1231). The resulting plasmid, named, pNG-Tn-*pyrF*, was used for *in vitro* transposition. *E. coli* DH5α cells were transformed by chemical transformation. Plasmid DNA was isolated from liquid cultures by using QIAprep spin miniprep columns (Qiagen). The plasmid constructs were confirmed by restriction analysis and Sanger sequencing. The full sequence of pNG-Tn-*pyrF* is available upon request.

### MarC9 transposase purification and *in vitro* transposition of *P. furiosus* genomic DNA

MarC9 transposase was purified according to a previously published method^39^. Briefly, an *E. coli* DH5α λ*pir* strain bearing a plasmid encoding an inducible gene coding for MarC9 (hyperactive Mariner transposase) fused to the Maltose-Binding Protein (MBP) was cultivated in LB, and the overexpression of MBP-MarC9 was induced for 2 hours by addition of IPTG (isopropyl β-D-1-thiogalactopyranoside) during the exponential phase. Cells were lysed by sonication, and the lysate was applied to a column of amylose resin. MBP-MarC9 was eluted by addition of a maltose-containing buffer, aliquoted and stored at −80 °C. A typical purification yielded 100 μg of purified MBP-MarC9 at a concentration of 500 μg/ml. Transposition reactions were also performed as described, with minor modifications^31^,^39^,^40^. The transposition reactions were performed in a buffer containing 10% glycerol, 25 mM HEPES pH 7.9, 250 μg purified and heat-inactivated bovine serum albumin, 2 mM DTT, 100 mM NaCl and 5 mM MgCl_2_. The optimal concentration of MarC9 for the reaction was determined empirically by performing transposition reactions with 1 μg genomic DNA, 1 μg of pNG-Tn-*pyrF* plasmid, and a range of two-fold increasing transposase concentrations, followed by 2 hours of incubation at 30 °C. The reaction products were separated by gel electrophoresis and visualised with ethidium bromide staining. Activity of the transposase can be assessed by analysing conversion of the plasmid from the supercoil form into three other forms: nicked (one or both IRs cut on a single strand), linear (double-strand break in a single IR) and shortened linear (both IR cuts and totally excised transposon) (Fig. 2B). Observation of those three forms have been previously described, and longer incubation time did not modify their relative abundances. High concentrations of transposase do not improve the abundance of plasmid backbones with the transposon totally excised, but induce an apparent degradation of gDNA, probably because of a non-specific activity of the transposase on IR-like sites in the chromosome. In contrast to protocols for purification of native MarC9 transposase from inclusion bodies^40^, no nuclease contaminating activity was observed with purified MBP-MarC9.

Transposition of genomic DNA for natural transformation was performed on 10 μg of plasmid and 10 μg of gDNA. Buffer and transposase volumes were adjusted accordingly. After transposition, the reaction was inactivated by heating at 75 °C for 10 min, then DNA was purified by phenol-chloroforme extraction and ethanol precipitation. The transposition junctions in the DNA were repaired as previously described by filling the junctions with T4 DNA polymerase and nick-repairing with *E. coli* DNA ligase.

### Transformation of *P. furiosus* with transposon-mutagenised DNA

Transposed and repaired DNA was used for natural transformation in *P. furiosus* strain JFW002. Overnight culture was grown to ~10^8^ cells/ml (as determined by counting in a Thoma counting chamber) in minimal liquid medium with uracil. 2 to 4 mL of culture were cooled on ice and centrifuged at 4000 rpm for 10 min. The cell pellet was resuspended in 100–200 μl 1X base salts (1/20 of the original culture volume). The cell suspension was mixed with transposed DNA in a proportion of 1 μg DNA for 10^8^ cells. Approximately 2 to 4 μg of DNA were used in each transformation experiment. The mixtures were spread on the selective solid medium without uracil using 100 μl of mixture per plate with glass beads.

### Southern-blot analysis of transposition mutants

Approximately 1 μg of gDNA was digested with *Hind*III restriction enzyme (Thermo Scientific) and resolved on a 0.8% agarose gel for 1 hour at 80 V. Genomic DNA fragments were transferred from the gel to a Hybond-N + membrane (Amersham) using alkaline capillary transfer. The membrane was blocked for 1 hour at 42 °C in ECL Gold Hybridisation Buffer (Amersham) supplemented with 0.5 M of NaCl and 5% (w/v) of single strand salmon sperm DNA. Hybridisation was performed at 42 °C overnight in ECL Gold Hybridisation Buffer, hybridisation probe was labelled by ECL Direct Labelling and Detection System kit (Amersham). Washing, labelling and detection were accomplished according to the manufacturer’s directions using the ECL Direct Labelling and Detection System kit (Amersham). The probe was produced via PCR of plasmid DNA pNG-Tn-*pyrF* using primers pyrF-1_F and pyrF-2_R (Supplementary Table S3).

### Identification of transposition insertion sites

PCR amplification to confirm the presence of transposon insertion was performed using the primers pyrF_1_F and pyrF_2_R (Supplementary Table S3). Single Primer Colony PCR was used to identify the sites of transposon insertion^33^. PCR was performed as follows: initial denaturation of genomic DNA of tested mutants (98 °C, 5 min), 20 high stringency cycles (98 °C, 10 sec; 50 °C, 30 sec; 72 °C, 90 sec), 30 low stringency cycles (98 °C, 10 sec; 30 °C, 30 sec; 72 °C, 90 sec) and 30 high stringency cycles (98 °C, 10 sec; 50 °C, 30 sec; 72 °C, 90 sec) followed by a final extension at 72 °C for 7 min. Primer *pyrF*_P1 (Supplementary Table S3) is designed in order to hybridise 118 base pairs upstream of the inverted tandem repeat (ITR). A second round of PCR is then performed using a ten-fold dilution and the above PCR reaction with primer pyrF_P2 (Supplementary Table S3) which hybridises 20 pairs downstream of pyrF_P1. This PCR reaction was send out for Sanger sequencing (GATC-biotech, Germany) using primer pyrF_seq.

## Additional Information

**How to cite this article**: Guschinskaya, N. *et al*. Random mutagenesis of the hyperthermophilic archaeon *Pyrococcus furiosus* using *in vitro* mariner transposition and natural transformation. *Sci. Rep.* **6**, 36711; doi: 10.1038/srep36711 (2016).

**Publisher’s note:** Springer Nature remains neutral with regard to jurisdictional claims in published maps and institutional affiliations.

## Supplementary Material

Supplementary Information

## Figures and Tables

**Table 1:** Positions of transposon insertion in the genome of *P. furiosus* COM1, with corresponding genes, proteins and their annotation.

**Mutant strain**	**Transposon insertion coordinates in COM1**	Gene*	**Protein**	**Annotation**
1	116279	PF0300	AFN03089.1	hypothetical protein GTP: adenosyl-cobinamide-phosphate guanylyltransferase
2	869063	PF1118	AFN03909.1	CRISPR-associated protein Cas1
3	216315	PF0407	AFN03198.1	hypothetical protein
4	1200285	PF1504	AFN04287.1	large helicase-like protein COG1201 Lhr-like helicases
5	1770485	PF1756	AFN04884.1	hypothetical protein COG0393 Uncharacterized conserved protein
6	775060	PF1016	AFN03812.1	2-hydroxyhepta-2,4-diene-1,7-dioate isomerase
7	482399	PF0669	AFN03462.1	hypothetical protein COG0454 Histone acetyltransferase HPA2 and related acetyltransferases
8	811244	PF1055	AFN03851.1	threonine synthase
9	1526411	PF2033	AFN04610.1	hypothetical protein predicted Fe-S oxidoreductase of SAM family
10	737427	PF0978	AFN03773.1	hypothetical protein COG1800 Predicted transglutaminase-like proteases
11	982898	PF1245	AFN04032.1	d-nopaline dehydrogenase COG0446 Uncharacterized NAD(FAD)-dependent dehydrogenases
12	1489490	PF0006	AFN04032.1	ABC transporter COG1131 ABC-type multidrug transport system, ATPase component

^*^The corresponding gene loci numbers from the wild-type DSM 3638 strain are presented for convenience.
