# Supplementary material for: Random mutagenesis of the hyperthermophilic archaeon Pyrococcus furiosus using in vitro mariner transposition and natural transformation
Source: Sci Rep. 2016 Nov 8;6:36711. doi: 10.1038/srep36711 (PMC5099854; doi:10.1038/srep36711)
Supplement: Supplementary Information [file srep36711-s1.pdf]

Supplementary Material

Random mutagenesis of the hyperthermophilic archaeon *Pyrococcus furiosus* using in vitro mariner transposition and natural transformation

Natalia Guschinskaya[1,2,3], Romain Brunel[1,2], Maxime Tourte[3,5], Gina L. Lipscomb[4], Michael W. W. Adams[4], Philippe Oger[3,5]*, Xavier Charpentier[1,2]*

Supplementary Table S1. Microbial strains used in the work.

Strain	Genotype	Reference
P. furiosus DSM 3638	Wild type	[1]
P. furiosus COM1	Δ*pyrF*	[2]
P. furiosus JFW002	Δ*pyrF* Δ*trpAB*	[3]
E. coli DH5α	F− *endA1 supE*44 *thi-1 recA1 relA1 gyrA96 deoR nupG* 80*lacZ* Δ*M15* Δ(*lacZYA-argF*)*U169 hsdR17* (*rk*$^{-}$ *mk*$^{+}$)	Invitrogen

Supplementary Table S2. Plasmid used in this work

Plasmid	Genotype	Reference
pJFW070	P*gdh-pyrF* cassette, containing 283-bp portion of the intergenic region upstream of *gdh* (PF1602) joined with *pyrF* gene and T1 terminator from the histone gene *hpyA1* (PF1722)	[3]
pMalC9	Plasmid for expression and purification of mariner transposase fused to the maltose binding protein. Carries the ampicillin resistance gene.	[4]
pJET1.2/blunt	Positive selection cloning vector from CloneJET PCR Cloning Kit #K1231	Thermo Scientific
pNG-Tn-*pyrF*	P*gdh-pyrF* cassette from pJFW070 was amplified with Tn_pyrF_for and Tn_pyrF_rev primers, and the PCR product was cloned in pJET1.2/blunt	This work

Supplementary Table S3. Primers used in this work

Primer	Sequence (5' to 3')
pyrF_500_for	GAAAACAGATATCCGAAATAC
pyrF_500_rev	CTTGCGGTTATCTCCCATATC
pyrF_1000_for	GAAATCCTCTACTTTCTTGAC
pyrF_1000_ref	GAGTTGTGTCAAGAGGATG
GL061	CTCAACTGTGATGTTTGTCTTGC
GL062	CGTTGGCAAACAACTTCCTG
pyrF_2000_for	GGTTTCTGAATACTCTATTAAAG
pyrF_2000_rev	CCAGTTCCAATAAGACTAC
pyrF_3000_for	CTCAGGGAGCTTCTTAATTG
pyrF_3000_rev	GTGTAACTTCGCCCTCAAAG
Tn_pyrF_for	GATTACAGGTTGGATGATAAGTCCCCGGTCTGCTAATTAATTGATTTTAGATTGAAAATGGAGTGAGC
Tn_pyrF_rev	GATTACAGGTTGGATGATAAGTCCCCGGTCTACTAATTAATTGATTTTAGCCTGCAGGTCCCCCTAAAAAAG
pyrF_1_F	TGTACTAGCGTTGGACGTGT
pyrF_2_R	AGCTCCATTCTTTCACCTCCTC
pyrF_P1	CATCATCATTGGAAGGGCCA
pyrF_P2	GGGAAGCCGCTAAGAAGATT
pyrF_seq	GGAGGTGAAAGAATGGAGCTCA

1. Fiala, G. & Stetter, K. O. Pyrococcus furiosus sp. nov. represents a novel genus of marine heterotrophic archaebacteria growing optimally at 100°C. *Arch. Microbiol.* **145,** 56–61
2. Lipscomb, G. L. *et al.* Natural competence in the hyperthermophilic archaeon Pyrococcus furiosus facilitates genetic manipulation: construction of markerless deletions of genes encoding the two cytoplasmic hydrogenases. *Appl. Environ. Microbiol.* **77,** 2232–2238 (2011).
3. Farkas, J. *et al.* Recombinogenic properties of Pyrococcus furiosus strain COM1 enable rapid selection of targeted mutants. *Appl. Environ. Microbiol.* **78,** 4669–4676 (2012).
4. Akerley, B. J. & Lampe, D. J. Analysis of gene function in bacterial pathogens by GAMBIT. *Methods Enzymol.* **358,** 100–108 (2002).

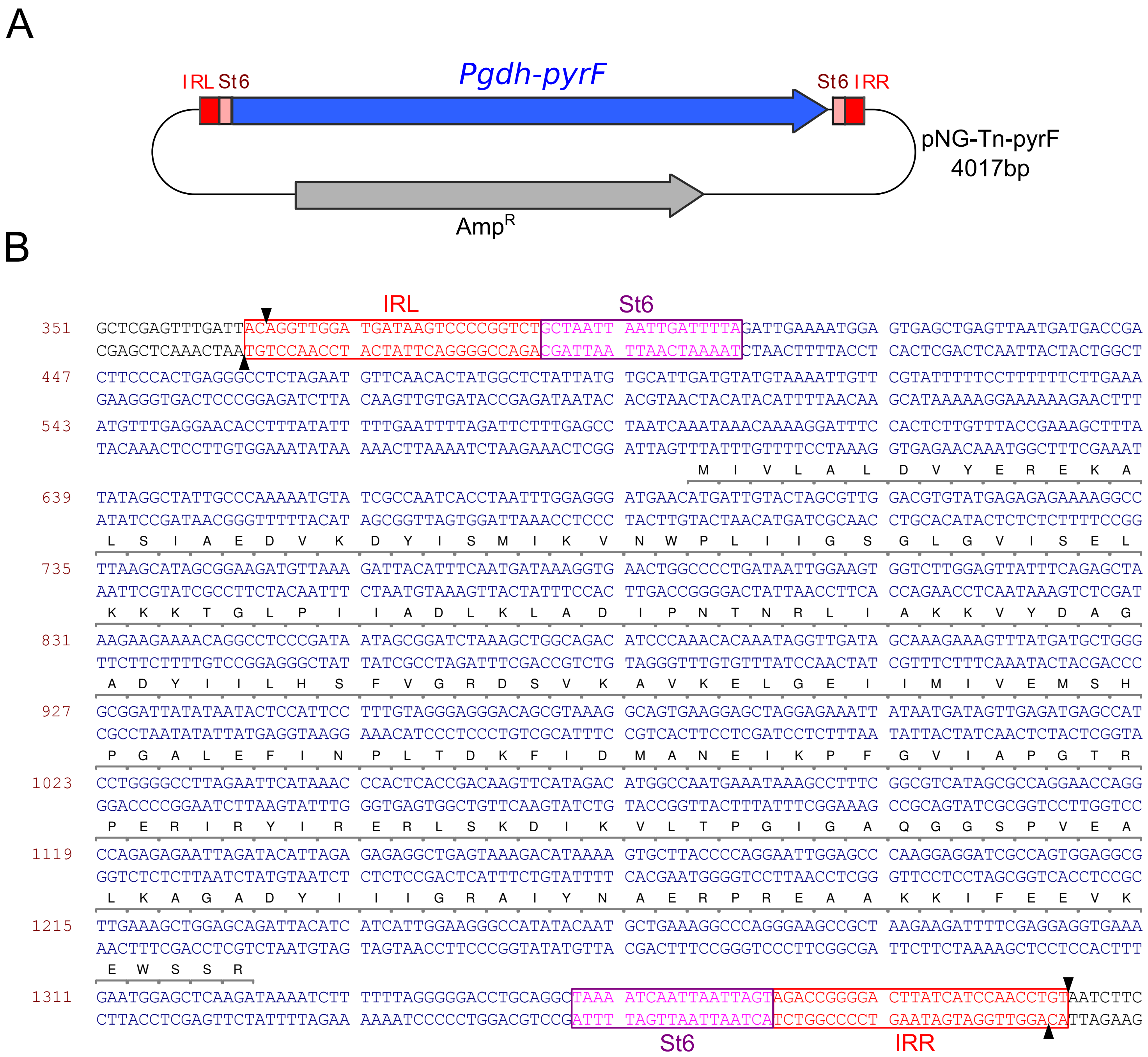

Supplementary figure S1. Schematic representation of pNG-Tn-pyrF plasmid carrying the Pgdh-pyrF transposable element. A) The composite gene made of the *gdh* promoter and the coding sequence of *pyrF* is flanked by inverted repeats subtrates of the MarC9 transposase. Each inverted repeat (IRL, IRR) is preceded by a St6 sequence which carries stop codons in each of the 6 possible translation frame. B) Sequence of the transposable element. Black diamonds indicate the cleavage site of the MarC9 transposase within the inverted repeats.

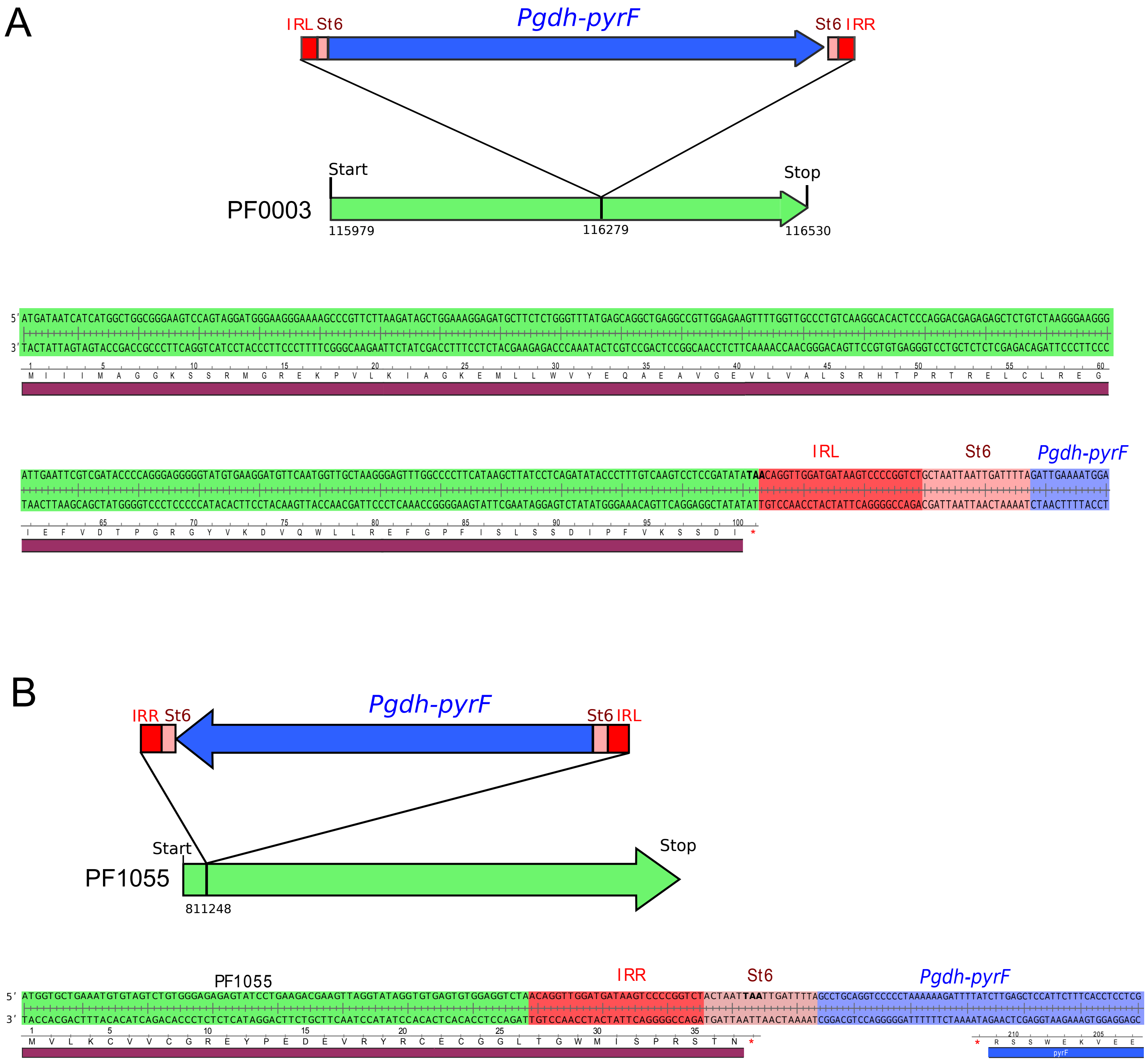

Supplementary figure S2. Schematic representation of transposon insertion obtained in the genes PF0003 (A) and PF1055 (B). In each case, insertion of the *Pgdh-pyrF* transposon results in truncation of the open reading frame by a premature stop codon (bold). The premature stop codons are either the result of the insertion of the inverted repeat IRL (A) or provided by the St6 sequence (B). IRL, inverted repeat left; IRR, inverted repeat right; St6, synthetic sequence introducing stop codon in each of the 6 possible reading frame.
